# Polyphenolic Fingerprint, Inter-Population Variability and Botanical Authentication of Wild *Polygonum aviculare* L. from Northwestern Romania

**DOI:** 10.3390/molecules31183237

**Published:** 2026-09-13

**Authors:** Iulia Ana Pasca, Ioana Larisa Bene, Daniela Gitea, Mihai Octavian Botea, Maria Flavia Gîtea, Marc Cristian Ghitea, Manuela Bianca Pasca

**Affiliations:** 1 Faculty of Medicine, “Iuliu Hațieganu” University of Medicine and Pharmacy, 400010 Cluj-Napoca, Romania; iulika_pasca@yahoo.com; 2 Department of Agriculture, Horticulture, Faculty of Environmental Protection, University of Oradea, 410048 Oradea, Romania; larisa.bene2002@gmail.com; 3 Department of Pharmacy, Faculty of Medicine and Pharmacy, University of Oradea, 410028 Oradea, Romania; bpasca@uoradea.ro; 4 Doctoral School of Biomedical Sciences, University of Oradea, 410087 Oradea, Romania; 5 Department of Surgical Disciplines, Faculty of Medicine and Pharmacy, University of Oradea, 410028 Oradea, Romania; 6 Faculty of Medicine and Pharmacy, University of Oradea, 410028 Oradea, Romania; gitea.mariaflavia@student.uoradea.ro (M.F.G.); ghitea.marccristian@student.uoradea.ro (M.C.G.)

**Keywords:** *Polygonum aviculare* L., phenolic compounds, flavonol glycosides, avicularin, HPLC-DAD-ESI-MS, wild medicinal plants, chemotaxonomic marker, Romania

## Abstract

*Polygonum aviculare* L. (PAV) is a widely distributed medicinal plant traditionally used for its diuretic, anti-inflammatory, and antioxidant properties, whose phenolic composition varies with provenance. This study provides the first integrated pharmacobotanical and phytochemical characterization of five wild PAV populations collected across a lowland-to-mountain gradient (105–605 m a.s.l.) in Bihor County, northwestern Romania. Botanical identity was authenticated by macroscopic and microscopic pharmacobotanical examination, which revealed a consistent anatomical organization alongside a qualitative morphological gradient related to altitude. The phenolic profile of the aerial parts was determined by HPLC-DAD-ESI-MS, and differences among populations were assessed by one-way ANOVA with Tukey’s HSD test. Fourteen phenolic compounds were detected and annotated, comprising one hydroxycinnamic acid, twelve flavonol glycosides, and one flavonol aglycone, with kaempferol derivatives predominating across all populations. Chlorogenic acid and rutin were confirmed using authentic reference standards, whereas the remaining compounds were tentatively annotated. A quercetin-O-arabinoside tentatively assigned as avicularin was consistently detected across all populations, supporting its potential as a candidate phytochemical marker. The sum of chromatographically quantified phenolic compounds varied markedly, showing a nearly six-fold difference between the richest and poorest populations, while the qualitative fingerprint remained conserved. Exploratory analysis indicated a general tendency toward lower phenolic content along a lowland-to-mountain environmental gradient characterized by increasing altitude and precipitation and decreasing mean annual temperature, although the total content was not significantly correlated with altitude. This pronounced inter-population variability underlines that wild-harvested PAV cannot be assumed uniform, highlighting the need for standardized extracts and site-specific phytochemical documentation in its pharmaceutical use.

## 1. Introduction

Natural products remain a major source of bioactive molecules for drug discovery, yet their therapeutic and commercial value depends critically on rigorous chemical authentication, as chromatographic fingerprinting is widely recommended as a rational approach for the quality assessment of herbal raw materials [1]. Unlike single-molecule pharmaceuticals, herbal raw materials are inherently variable in composition, being influenced by genetic background, developmental stage, and environmental growing conditions [2,3]. This variability, combined with frequent cases of botanical misidentification or adulteration in the herbal supply chain, underscores the need for combined pharmacobotanical and phytochemical characterization as a prerequisite for reliable quality control and reproducible pharmacological research [4]. The use of medicinal plants and natural remedies is deeply rooted in the historical development of medical and pharmaceutical practice [5], and many traditionally used species continue to attract contemporary pharmacological and phytochemical interest.

*Polygonum aviculare* L. (common knotweed, known in Romanian folk medicine as troscot) is an annual to perennial herbaceous species of the family Polygonaceae, widely distributed across temperate regions of Europe and Asia and commonly found as a ruderal plant along paths, roadsides, and disturbed soils. It has a long history of traditional use, with different plant parts employed as anthelmintic and antidiabetic agents and in the treatment of kidney disorders and skin conditions [6]. More recently, pharmacological interest in the species has extended beyond its traditional applications toward both its bioactivities and its formulation. Bioactivity-guided work on PAV aerial parts has identified its flavonol glycosides, together with quercetin and related aglycones, as strong inhibitors of α-glucosidase, with an inhibitory potency reported to exceed that of the reference drug acarbose [6], underscoring the pharmacological relevance of the flavonol fraction. In parallel, PAV extract has been incorporated into cell-penetrating-peptide-conjugated liposomes to enhance its transdermal delivery, with the conjugated carriers showing greater skin permeation and improved in vivo efficacy than conventional liposomes [7], illustrating the translational potential of its constituents.

Phytochemically PAV is recognized primarily as a source of flavonoid glycosides, predominantly derivatives of quercetin and kaempferol, alongside hydroxycinnamic acids such as chlorogenic acid [2,8]. A comprehensive phytochemical investigation of the species led to the isolation of 21 compounds, including fourteen flavonoids, underscoring its chemical richness and chemotaxonomic relevance within the genus [9]. Quercetin-3-O-arabinoside (avicularin) is frequently reported as a characteristic constituent of the species and has been proposed as a potential chemotaxonomic marker [8,9].

The pharmaceutical relevance of the species is further reflected in its recognition as a traditional herbal medicinal product: the European Medicines Agency’s Committee on Herbal Medicinal Products (HMPC) has issued an assessment report on PAV herba, in which the comminuted herbal substance is documented for oral use as a herbal tea [10]. The same report indicates a total flavonoid content in the range of 0.1–1% (occasionally up to 3%), dominated by kaempferol, quercetin and myricetin derivatives, with avicularin reported at approximately 0.2% [10], a wide reported range that itself points to the marked compositional variability addressed in the present study.

The biosynthesis and accumulation of these secondary metabolites are known to be strongly influenced by environmental factors, including altitude [11], temperature, and UV radiation exposure [12,13], which can lead to substantial chemical variability between geographically distinct populations of the same species [14]. This geographic variability poses a significant challenge for the standardization of herbal raw material, as the phytochemical profile, and therefore the potential bioactivity, of wild-harvested plant material cannot be assumed to be consistent across collection sites [1,4,14].

To the best of our knowledge, no comprehensive pharmacobotanical and phytochemical characterization of PAV populations from Romania has been reported [6,8,14]. The present study therefore aimed primarily to authenticate five wild PAV populations from northwestern Romania by macroscopic and microscopic pharmacobotanical examination, to characterize their phenolic fingerprint by HPLC-DAD-ESI-MS, to assess inter-population variability in phenolic content, and to evaluate selected flavonol glycosides as candidate markers for quality assessment. As the populations were distributed across a lowland-to-mountain gradient, we additionally explored, on a preliminary basis, whether their phenolic content co-varied with this environmental gradient. Given the small number of populations and the intrinsic covariation of altitude with temperature, precipitation, and soil type, this latter analysis was intended as an exploratory observation rather than a formal test of altitude as an independent driver of phenolic biosynthesis.

## 2. Results

### 2.1. Botanical Authentication and Sampling Characteristics

**Figure 1:**
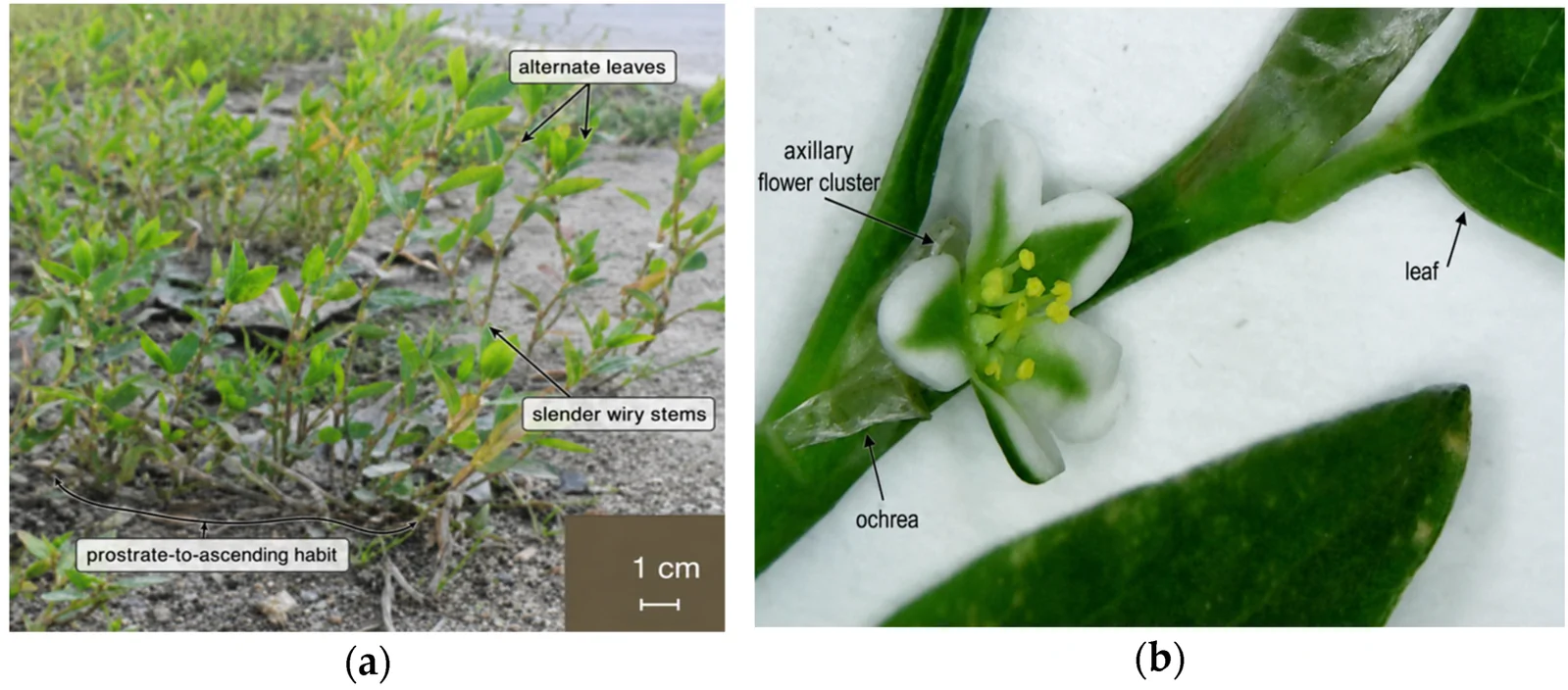

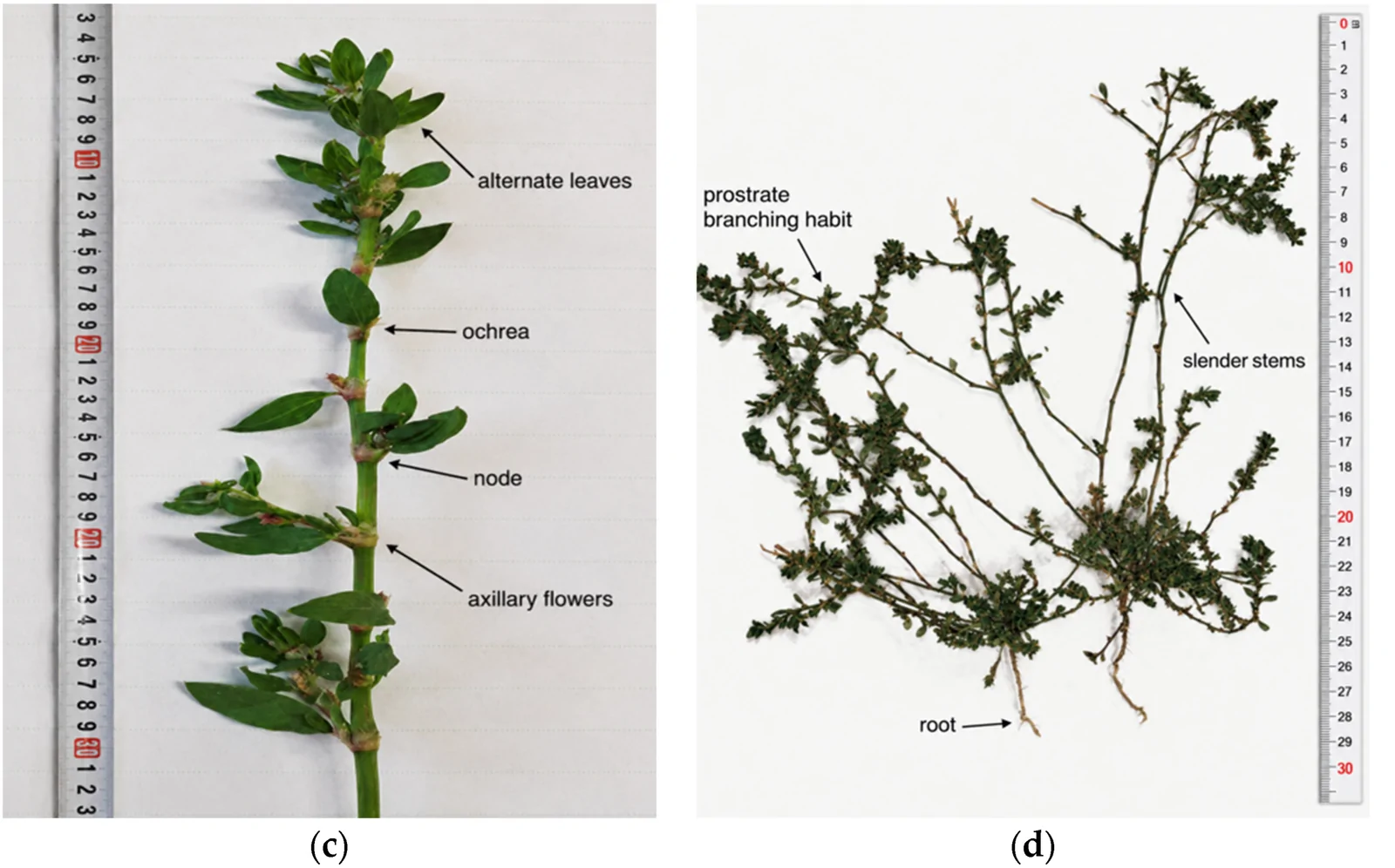
Macroscopic characterization of *Polygonum aviculare* L. (PAV): (**a**) habit in natural environment; (**b**) magnified detail of axillary flower cluster and ochrea; (**c**) nodal detail showing leaf arrangement and ochreae; (**d**) whole pressed specimen with scale.

Five populations of *Polygonum aviculare* L. (PAV1–PAV5) were collected from distinct locations in Bihor County, northwestern Romania, spanning an altitudinal range of 105–605 m a.s.l. Species identity was confirmed macroscopically, following the diagnostic characters described in Section 4 (Materials and Methods). All specimens displayed the typical prostrate-to-ascending habit of the species, with slender, wiry stems, alternate elliptic-to-lanceolate leaves, and characteristic silvery, membranous ochreae at each node (Figure 1). A morphological gradient was observed along the collection sites: specimens from higher-altitude locations (PAV4–PAV5) developed taller, more elongated stems bearing a reduced number of leaves, whereas specimens from the lowest-altitude site (PAV1, Santăul Mare) displayed shorter, more densely leaved stems.

### 2.2. Microscopic Characterization

**Figure 2:**
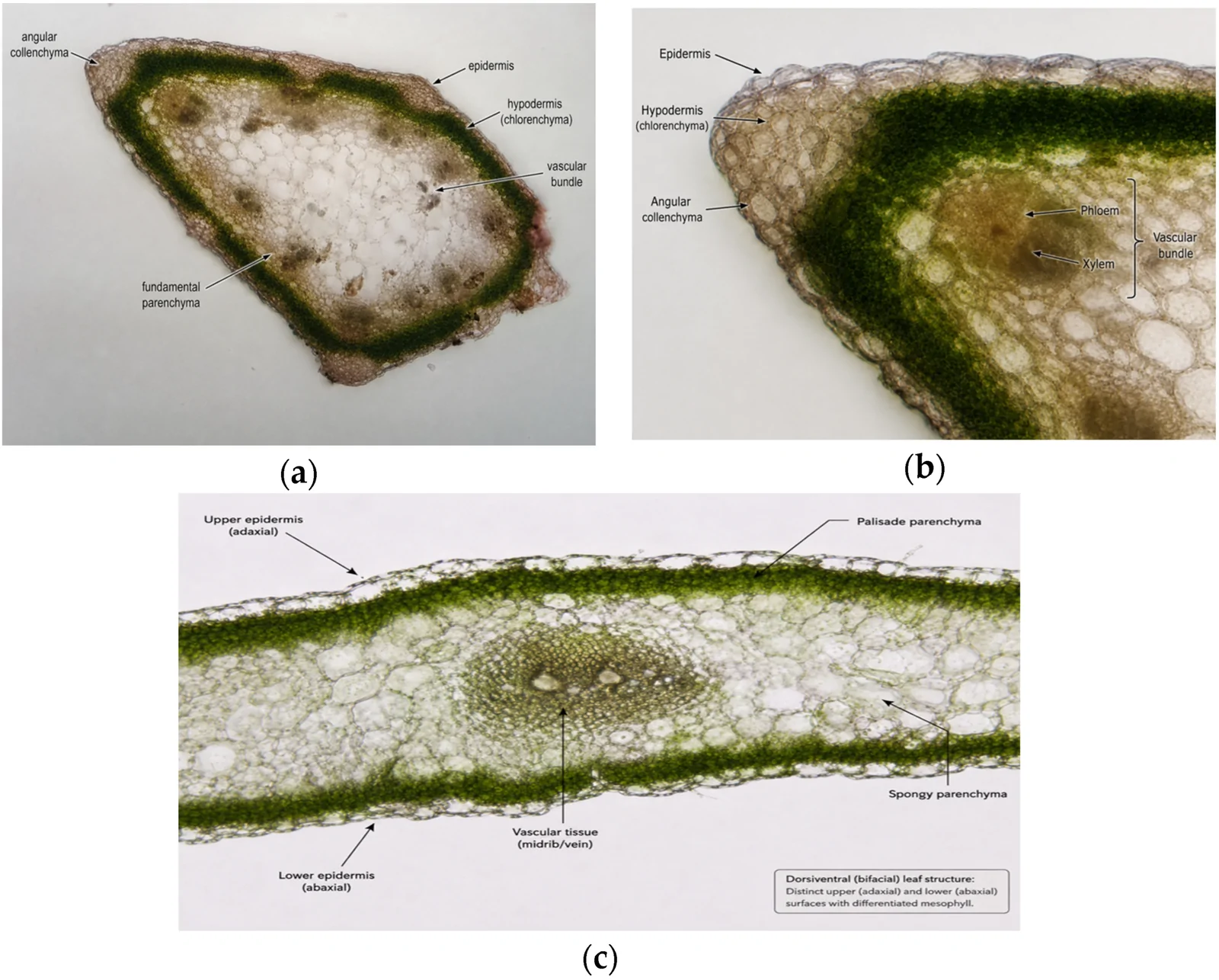
Transverse sections of *Polygonum aviculare* L. stem and leaf, stained with Genevez reagent: (**a**) stem cross-section, general view; (**b**) detail of angular collenchyma in the stem; (**c**) leaf cross-section.

Transverse sections through the stem and leaf of PAV, stained with Genevez reagent, revealed anatomical features consistent across the examined populations (Figure 2). In transverse section, the stem displayed an irregular, trapezoidal outline, with the following tissue sequence from the periphery inward: epidermis, hypodermis, fundamental parenchyma, and collateral vascular bundles. The epidermis consisted of a single layer of tightly packed cells with slightly convex outer walls. Immediately beneath it, the hypodermis comprised a well-defined chlorenchyma rich in chloroplasts, within which localized cell aggregations formed a collenchyma reinforced at the vascular bundle positions (angular collenchyma). The fundamental parenchyma, composed of thin-walled cellulosic cells staining red with Genevez reagent, contained the vascular bundles; each bundle was mixed (collateral), with phloem positioned externally and xylem toward the stem axis, and was flanked by two arcs of sclerenchymatous tissue, with the phloem-side arc being more developed (Figure 2a,b).

In transverse section, the leaf exhibited a bifacial (dorsiventral) structure, with a darker-colored upper (adaxial) surface and a lighter green lower (abaxial) surface. The upper epidermis consisted of a single cell layer, beneath which a chloroplast-rich palisade parenchyma of 2–3 layers of elongated, tightly packed cells was observed. The middle region of the lamina contained 3–4 layers of larger, polyhedral, less chlorophyll-rich cells forming the spongy parenchyma, within which the vascular tissue (midrib and secondary veins) was embedded (Figure 2c).

### 2.3. Phenolic Profiles of the Investigated Populations

Fourteen phenolic compounds were detected and annotated in the ethanolic extracts of PAV based on their retention behavior, UV-Vis absorption spectra, in-source fragmentation (loss of the glycosidic residue to yield the protonated aglycone) and comparison with authentic reference standards, published literature, and the Phenol-Explorer database (Table 1). Chlorogenic acid and rutin were confirmed using authentic reference standards, whereas the remaining compounds were tentatively annotated. The characterized profile comprised one hydroxycinnamic acid, twelve flavonol glycosides, and one flavonol aglycone, predominantly represented by quercetin and kaempferol derivatives. A quercetin-O-arabinoside tentatively assigned as avicularin was detected in all five populations, supporting its further evaluation as a candidate phytochemical marker for the species. The consistent detection of aglycone ions at *m*/*z* 303, 287, and 319, together with the characteristic neutral losses of the glycosidic residues, confirmed the quercetin, kaempferol, and myricetin backbones of the annotated flavonol glycosides [2,8,9].

**Figure 3:**
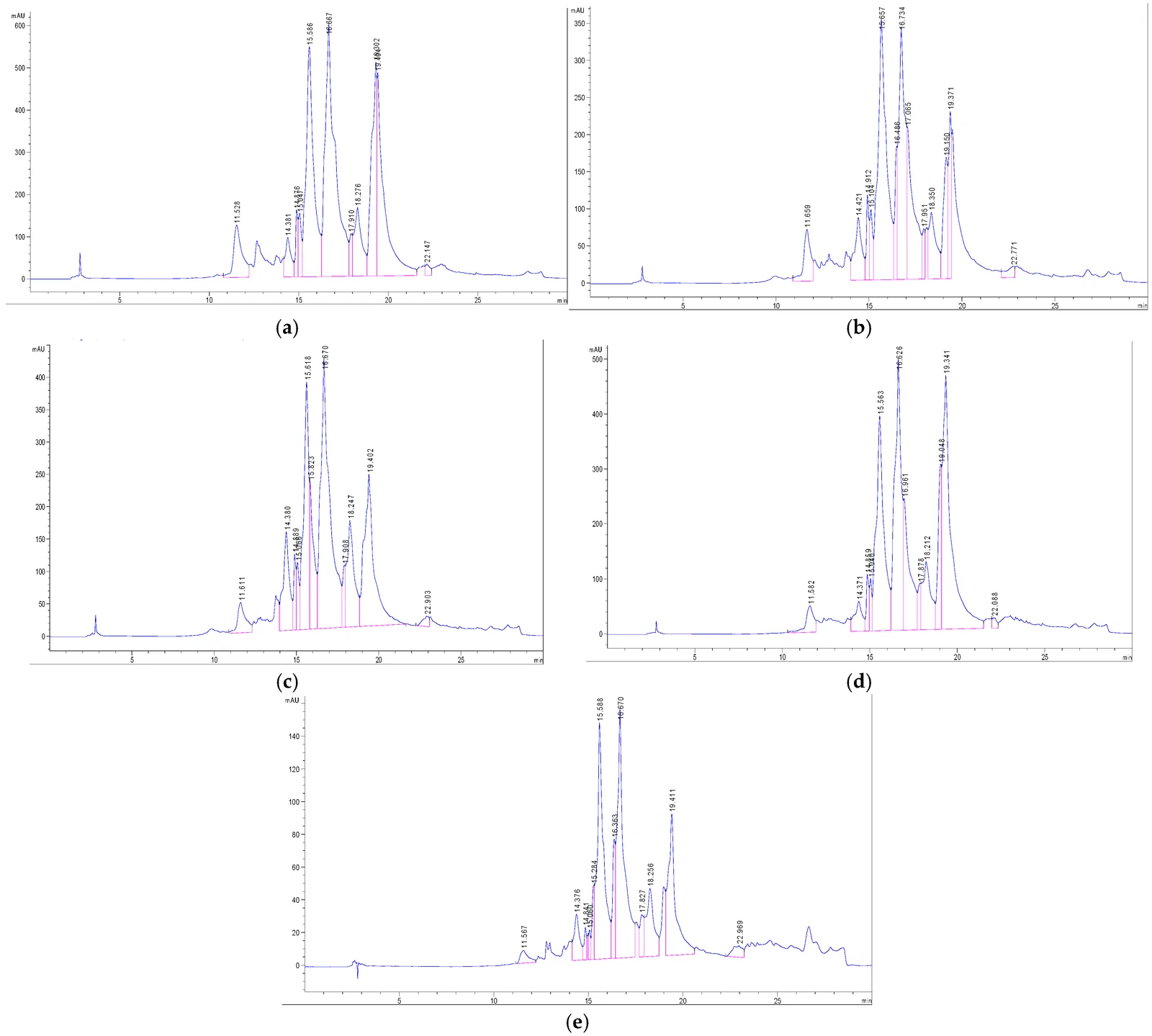
Representative HPLC-DAD chromatographic profiles (λ = 340 nm) of *Polygonum aviculare* L. ethanolic extracts: (**a**) PAV1 (Santăul Mare, 105 m); (**b**) PAV2 (Șoimi, 201 m); (**c**) PAV3 (Dumbrăvița de Codru, 380 m); (**d**) PAV4 (Izbuc, 515 m); (**e**) PAV5 (Băița, 605 m). Peak numbering corresponds to the compound identities listed in Table 1. The chromatograms correspond to individual analyses and were not mathematically averaged.

Representative HPLC-DAD chromatographic profiles (λ = 340 nm) of the five populations are presented in Figure 3, illustrating the overall similarity of the phenolic fingerprint across sites, alongside visible differences in relative peak intensities consistent with I confirm.the quantitative variation reported in Table 1.

The quantitative content of the fourteen phenolic compounds varied markedly among the five populations, and one-way ANOVA revealed statistically significant differences for all compounds (*p* < 0.05; *p* < 0.001 for thirteen of the fourteen compounds). The individual contents, expressed as mean ± standard deviation together with the corresponding Tukey HSD groupings, are presented in Table 2. The chromatographic sum of the fourteen quantified phenolic compounds ranged from 3.14 mg/g dry weight in PAV5 to 18.81 mg/g in PAV1, indicating a nearly six-fold difference between the richest and the poorest population. Kaempferol-O-malonylglucoside was the most abundant single compound, reaching 5.59 ± 0.02 mg/g in PAV1, followed by kaempferol-O-glucuronide (up to 2.65 ± 0.01 mg/g in PAV4) and quercetin-O-arabinoside (up to 2.05 ± 0.02 mg/g in PAV1). PAV1 and PAV4 were consistently characterized by the highest levels of most flavonol glycosides, whereas PAV5 showed the lowest content for nearly all compounds. This pronounced quantitative variability, observed despite a qualitatively uniform phenolic profile, is consistent with the substantial inter-population phytochemical variability previously reported for PAV [14].

### 2.4. Distribution of Phenolic Subclasses

To describe the overall phytochemical pattern of the investigated populations, the fourteen identified compounds were grouped into four subclasses according to their core structure: hydroxycinnamic acids (chlorogenic acid), quercetin glycosides, kaempferol derivatives, and myricetin glycosides. The absolute content and relative contribution of each phenolic subclass to the chromatographic sum quantified in each population are summarized in Table 3.

**Figure 4:**
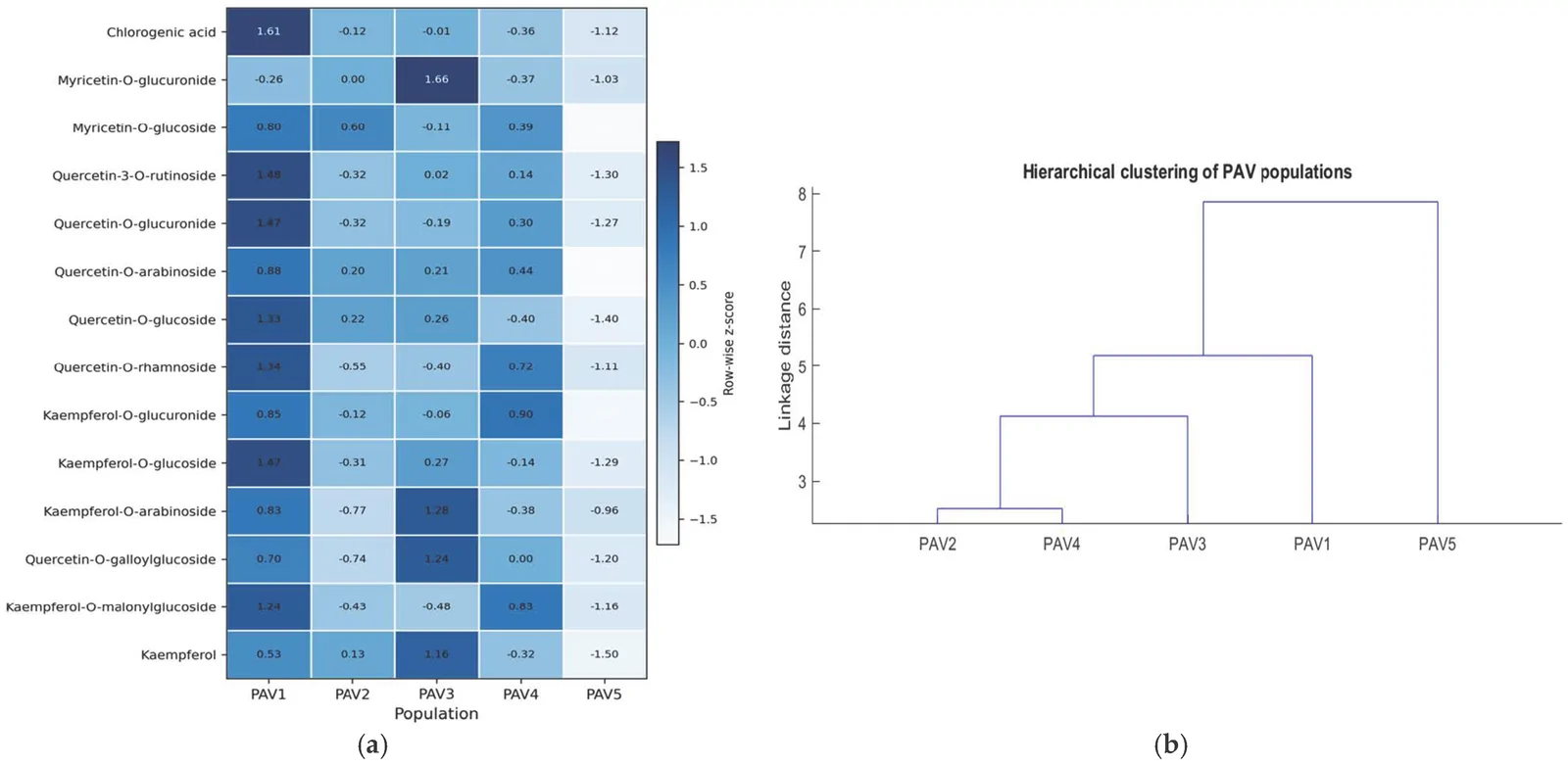
Comparative visualization of the phenolic profiles of the five *Polygonum aviculare* L. populations (PAV1–PAV5): (**a**) heatmap of row-wise standardized (z-score) contents of the fourteen phenolic compounds; darker shades indicate above-average and lighter shades below-average relative content within each compound; (**b**) hierarchical clustering dendrogram based on the standardized phenolic profiles, using Euclidean distance and Ward’s linkage method. Values used for the analyses are means (*n* = 3 biological replicates).

To obtain a broader overview of the phytochemical composition and to compare the five populations independently of the absolute concentration of each compound, the individual phenolic contents were standardized row-wise (z-scores) and visualized as a heatmap (Figure 4). This representation allows the relative accumulation pattern of each compound to be compared across populations on a common scale, thereby revealing population-specific trends that would otherwise be masked by the large differences in absolute content among compounds.

The heatmap revealed a clear population gradient. PAV1 was characterized by predominantly above-average levels across nearly all compounds, confirming its status as the richest population, whereas PAV5 displayed consistently below-average values, in agreement with its lowest chromatographic sum of quantified phenolic compounds. PAV2 and PAV4 showed intermediate, more balanced profiles. Beyond this overall gradient, several compounds exhibited distinct population-specific accumulation patterns: myricetin-O-glucuronide reached its highest relative level in PAV3, while kaempferol-O-arabinoside and quercetin-O-galloylglucoside were also markedly enriched in this population. Hierarchical clustering supported these differences, showing the closest similarity between PAV2 and PAV4, followed by PAV3, whereas PAV5 displayed the most distinct overall phenolic profile. These localized enrichments indicate that, despite a qualitatively uniform phenolic fingerprint, the five populations retain a certain degree of chemical individuality in the relative proportions of specific flavonol glycosides.

### 2.5. Exploratory Relationship Between Phenolic Content and the Environmental Gradient

Because the five populations of PAV were distributed along a lowland-to-mountain gradient, we explored on a preliminary basis whether their phenolic content co-varied with this environmental gradient. This analysis should be interpreted with caution: with only five populations and altitude intrinsically confounded with mean annual temperature, precipitation, and soil type, it is statistically underpowered and cannot isolate altitude as an independent factor. Spearman rank correlations between altitude and both the chromatographic sum of quantified phenolic compounds and the four phenolic subclasses are summarized in Table 4.

The chromatographic sum of quantified phenolic compounds was not significantly correlated with altitude (Table 4). All correlation coefficients were negative, suggesting a general tendency toward lower phenolic content at higher-altitude, cooler, and more humid sites, but none reached significance except the hydroxycinnamic fraction, represented solely by chlorogenic acid. As five subclasses were tested without correction for multiple comparisons, this single nominally significant result should be regarded as tentative and may reflect a chance finding. The non-monotonic distribution of total content along the gradient—with PAV1 (lowland) the richest and PAV5 (highest altitude) the poorest, while intermediate populations departed from a strictly linear pattern—further argues against a simple altitudinal cline. Because altitude was strongly collinear with temperature and precipitation, correlations with these variables mirrored those with altitude and are not reported separately. These results are therefore exploratory rather than confirmatory.

## 3. Discussion

The phenolic profile obtained for PAV in the present study, dominated by flavonol glycosides of quercetin and kaempferol, together with chlorogenic acid as the sole hydroxycinnamic acid, is in close agreement with previous phytochemical investigations of the species. In a comprehensive study of the whole plant, Yu et al. isolated twenty-one constituents, of which fourteen were flavonoids, with kaempferol and its derivatives and quercetin glycosides among the predominant structures [8,9]. The prevalence of quercetin- and kaempferol-based glycosides observed across our five populations mirrors this pattern and reinforces the view that these two flavonol classes constitute the phenolic backbone of PAV [8,15]. Notably, glycosylation at the C-3 position of the flavonol nucleus, reported as the most common structural feature of the genus *Polygonum* [9], is consistent with the C-3 arabinoside, glucoside, glucuronide, rhamnoside, and galloyl/malonyl derivatives detected in the present work.

A quercetin-O-arabinoside, tentatively assigned as avicularin (quercetin-3-O-arabinoside), was consistently detected in all five populations [9,15,16]. This is further reinforced by the recurrent isolation of avicularin from the aerial parts of PAV in independent phytochemical studies from different geographic origins, including Korean and Chinese material [9], which indicates that the compound is a stable constituent of the species rather than a locally determined feature. Its uniform occurrence across our populations therefore suggests that avicularin may serve as a candidate target for further validation in analytical authentication, independently of local growing conditions. Beyond authentication, the flavonol-rich composition documented here is of pharmacological relevance: recent bioactivity-guided work on the aerial parts of PAV identified quercetin, kaempferol, avicularin, and related aglycones as the principal contributors to its antioxidant, anti-inflammatory, and α-glucosidase-inhibitory effects, with kaempferol showing the strongest α-glucosidase inhibition and quercetin the strongest antioxidant and nitric-oxide-inhibitory activity [15,16]. The predominance of kaempferol and quercetin derivatives in the populations analysed here therefore suggests that Romanian wild PAV represents a promising source of these bioactive flavonols, although it should be noted that glycosylation has been shown to attenuate the activity of the parent aglycones [16,17].

With respect to the environmental gradient, phenolic compound levels generally tended to be lower at higher-altitude, cooler, and more humid sites. However, the chromatographic sum of quantified phenolic compounds was not significantly correlated with altitude, and only the hydroxycinnamic acid subclass, represented by chlorogenic acid, showed a nominally significant negative correlation. These results are therefore exploratory. The direction of altitude-related trends is not universal in the literature, as environmental factors such as temperature, UV radiation, water availability, and soil conditions can differentially affect secondary-metabolite accumulation [12,13]. Increased UV radiation and lower temperatures at higher elevations have been reported to stimulate phenolic accumulation in some species, such as Coleus forskohlii [11], whereas other studies describe the opposite pattern, with lower-altitude populations accumulating higher phenolic levels, generally attributed to more favourable soil fertility and water availability. Notably, a related study by our group on Glechoma hederacea L. from the same region found that the higher-altitude population (553 m) exhibited significantly higher total phenolic content than the lower-altitude one (261 m) [18]; the reverse of the tendency observed here. This divergence indicates that the altitudinal response of phenolic biosynthesis is species-specific and, in the present case, cannot be ascribed to altitude alone, since altitude co-varied with temperature, precipitation, and soil type along the sampling gradient. The observed pattern is thus best interpreted as reflecting the integrated environmental gradient rather than any single factor.

The marked quantitative variability among populations, set against a qualitatively conserved phenolic fingerprint, is consistent with earlier reports of pronounced phytochemical variability in PAV. In a comparative HPLC study of thirteen populations collected across thousands of kilometres of northern Asia, Petruk and Vysochina reported that the same qualitative set of flavonol glycosides, hyperoside, juglanin, avicularin, and quercitrin, was consistently present, yet the dominant compound differed between populations, with hyperoside and juglanin prevailing in some, avicularin in others, and quercitrin in a further group, leading the authors to conclude that PAV is characterised by a high biochemical variability in the composition and content of its phenolic compounds, without a clear dependence on the growing site [14]. Our findings extend this observation to a regional, altitudinal scale within a single county: although the qualitative fingerprint was conserved across all five Romanian populations, the quantitative content varied markedly, and the dominant compound shifted between kaempferol-O-malonylglucoside and kaempferol-O-glucuronide depending on the population.

This variability has direct implications for the pharmaceutical use of the species. Because the concentration of the bioactive flavonols, and therefore the potential therapeutic effect, cannot be assumed to be uniform across wild-harvested material, the use of raw plant material of unspecified origin introduces an inherent risk of batch-to-batch variability in any derived preparation. The nearly six-fold difference in the chromatographic sum of quantified phenolic compounds between the richest (PAV1) and the poorest (PAV5) population illustrates the magnitude of this risk. From a pharmaceutical standpoint, these results therefore argue in favour of the use of standardized (titrated) extracts, in which the content of a defined marker or class of markers is adjusted to a fixed value, rather than crude extracts of variable composition. In this context, avicularin and the total flavonol content emerge as suitable candidate markers for such standardization, supporting reproducible quality and potentially contributing to the consistency of the pharmacological properties of PAV-based preparations. This variability thus reinforces the need to document site-specific phytochemical profiles and to adopt standardized extracts prior to the pharmaceutical valorization of PAV as a herbal raw material, and suggests that collection-site selection may itself be a relevant determinant of the polyphenol yield of derived extracts [15,19].

The concurrent macroscopic morphological gradient, taller, more elongated stems bearing fewer leaves at higher-altitude sites, versus shorter, densely leaved stems at the lowest-altitude site (PAV1), may be tentatively interpreted in light of the growth–differentiation balance hypothesis, which posits a trade-off between resource allocation to vegetative growth and to secondary-metabolite (defence-related) biosynthesis [20,21,22]. Under this framework, the reduced leaf number accompanying lower phenolic content at higher altitudes could reflect a shift toward axial elongation at the expense of foliar surface area and associated phenolic investment. This interpretation remains speculative given the exploratory, qualitative nature of the morphological observations and would benefit from quantitative morphometric validation in future studies.

Finally, these findings are broadly consistent with previous studies on medicinal plants from Bihor County, which have highlighted the value of integrating pharmacobotanical authentication with phytochemical and phenolic profiling for the characterization and quality assessment of regionally sourced medicinal flora [18]. More broadly, chromatographic fingerprinting and quantitative phytochemical profiling are recognized as valuable tools for the standardization and quality control of herbal materials [19]. Taken together, the present results provide baseline pharmacobotanical and phytochemical data for wild PAV from northwestern Romania and underline the importance of combining botanical authentication with quantitative phenolic profiling in the quality assessment of this species.

Limitations. This study has several limitations that should be considered when interpreting the results. Quantification relied on authentic standards for only some compounds; the remaining flavonol glycosides were expressed as equivalents of the closest available standard, and three (compounds **6**–**8**) were only tentatively assigned without NMR confirmation. The small number of populations (*n* = 5) limited the statistical power, and no significant correlation was detected between the environmental gradient and the chromatographic sum of quantified phenolic compounds. Moreover, altitude was intrinsically collinear with temperature, precipitation, and soil type, so no single factor could be isolated. These findings should therefore be regarded as a basis for further studies using larger population sets and authenticated standards.

## 4. Materials and Methods

### 4.1. Plant Material and Botanical Authentication

**Figure 5:**
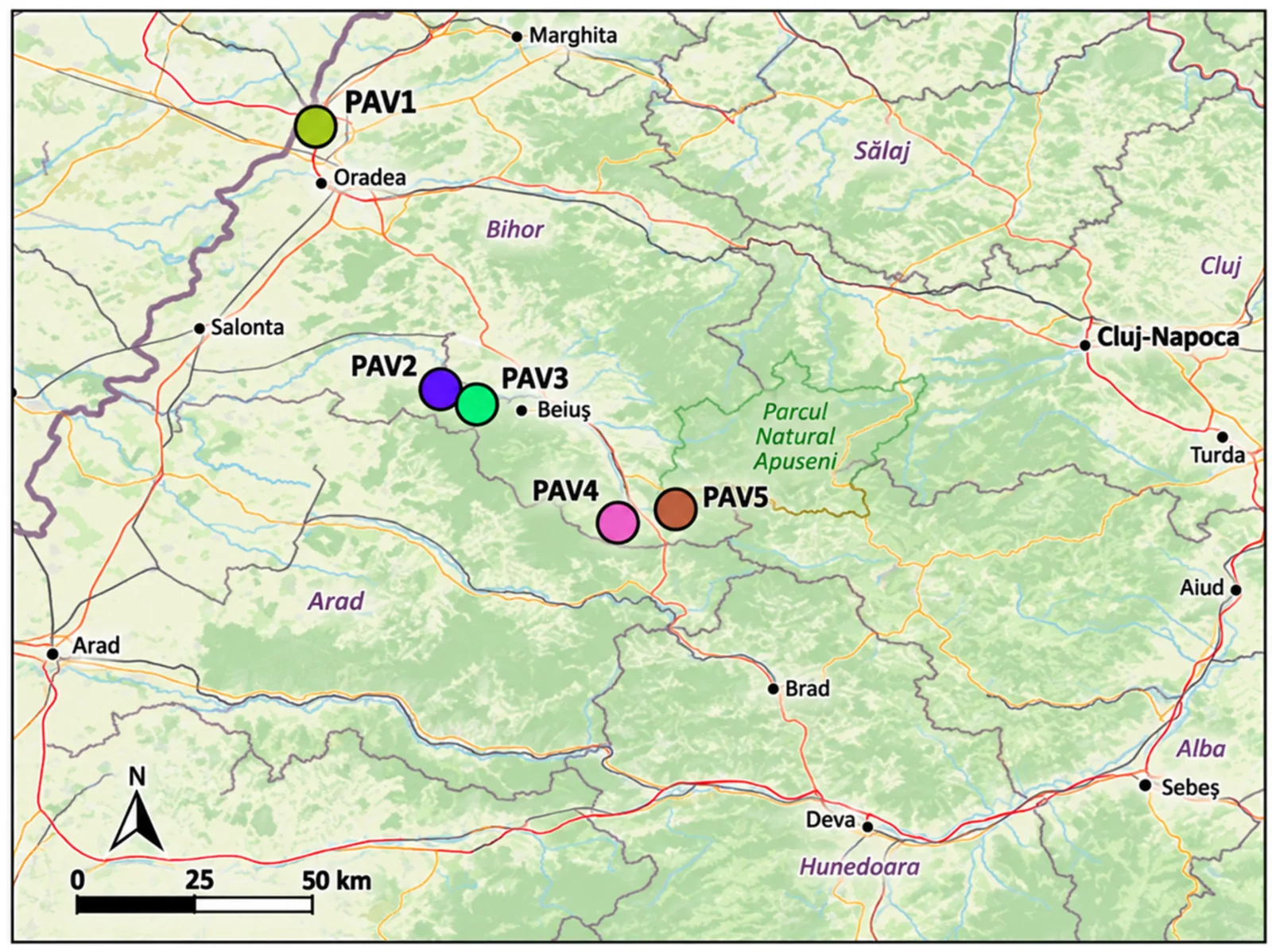
Geographical distribution of the five *Polygonum aviculare* L. sampling sites in Bihor County, northwestern Romania: PAV1-Santăul Mare; PAV2-Șoimi; PAV3-Dumbrăvița de Codru; PAV4-Izbuc; and PAV5-Băița. The map was generated using QGIS version 3.40.14, with OpenStreetMap used as the basemap (© OpenStreetMap contributors).

Five populations of *Polygonum aviculare* L. (PAV1–PAV5) were collected from distinct locations in Bihor County, northwestern Romania, during the flowering period [10], across an altitudinal range of 105–605 m a.s.l. The sampling sites were selected to represent successive positions along a regional lowland-to-mountain gradient rather than to maximize geographic separation. Accordingly, the five populations span contrasting altitudinal and pedoclimatic conditions within Bihor County. Although PAV2 and PAV3 are geographically relatively close, they occur at different elevations and represent distinct positions within the Codru-Moma hilly region. The geographical distribution of the sampling sites is presented in Figure 5, while their coordinates, altitudes, and pedoclimatic characteristics are summarized in Table 5. Current taxonomic nomenclature was checked against Plants of the World Online (POWO), facilitated by the Royal Botanic Gardens, Kew [23]. Botanical identity was authenticated by Manuela Bianca Pașca (Department of Pharmacy, Faculty of Medicine and Pharmacy, University of Oradea, Romania) on the basis of macroscopic pharmacobotanical examination, using pharmacopoeial diagnostic criteria from the Romanian Pharmacopoeia and the European Pharmacopoeia [24,25]. Voucher specimens (population codes PAV1–PAV5) were deposited in the Herbarium of the Faculty of Medicine and Pharmacy, University of Oradea, Romania, under accession numbers UOP 05.743–05.747.

### 4.2. Macroscopic, Microscopic and Morphological Characterization

The macroscopic characterization of specimens from the five *Polygonum aviculare* L. populations (PAV1–PAV5) was carried out in accordance with the recommendations of the 10th edition of the Romanian Pharmacopoeia [24], by observing the organoleptic characteristics of each plant part: external appearance (size, color, surface texture), smell, and taste. Dimensions were determined using a graduated ruler. The plant material was photographed using a Canon EOS R5 camera (Canon Inc., Tokyo, Japan), equipped with a Canon RF 35 mm F1.8 Macro IS STM lens (Canon Inc., Tokyo, Japan).

Microscopic preparations were obtained from fresh plant material by making transverse cross-sections of the stem and leaf, which were then stained with a hydroalcoholic solution of Genevez reagent (Congo red and chrysoidine) for 1–3 min [18], followed by repeated rinsing with distilled water to remove excess dye. Sections were examined and photographed using an Optika C-B10+ 24010 optical microscope (Optika, Ponteranica, Italy), equipped with 10×, 20×, and 40× objectives and an Optika B10 digital camera (Optika, Ponteranica, Italy), at the Laboratory of Pharmaceutical Botany, Faculty of Medicine and Pharmacy, University of Oradea.

### 4.3. Preparation of Plant Samples

At each sampling site, three individual *Polygonum aviculare* L. plants were randomly collected from spatially separated points during the flowering stage and considered independent biological replicates (*n* = 3 per population) [29]. Only healthy plants at a comparable phenological stage were selected [12]. The same aerial parts, including stems, leaves, and flowers, were collected from each individual. Each plant was maintained separately throughout drying, grinding, extraction, and phytochemical analysis. Repeated analytical measurements performed on the same extract were considered technical replicates and averaged to obtain a single value for each biological replicate.

### 4.4. Chemicals and Reagents

HPLC-grade acetonitrile and acetic acid were purchased from Merck KGaA (Darmstadt, Germany). Ultrapure water was obtained using a Direct-Q UV water purification system (Millipore, Burlington, MA, USA). Ethanol of analytical grade, used for extraction, was purchased from Chimreactiv SRL (Bucharest, Romania). Chlorogenic acid (purity ≥ 98%) and rutin (purity ≥ 99%), used as reference standards for chromatographic identification and quantification, were purchased from Sigma-Aldrich (St. Louis, MO, USA). All other reagents and solvents were of analytical or HPLC grade, as appropriate.

### 4.5. Extraction of Phenolic Compounds

A quantity of 0.5 g of dried, powdered plant material was extracted with 5 mL of 70% ethanol by vortex mixing for 1 min (Heidolph Instruments, Heidolph Instruments GmbH & Co. KG, Schwabach, Germany), followed by sonication for 30 min in an ultrasonic bath (Elma Schmidbauer GmbH, Singen, Germany) and centrifugation at 10,000 rpm for 10 min at 24 °C (Eppendorf AG, Hamburg, Germany) [30,31]. The supernatant was filtered through a 0.45 µm Chromafil Xtra nylon syringe filter (MACHEREY-NAGEL GmbH & Co. KG, Düren, Germany), and 20 µL of the filtrate was injected into the HPLC system.

### 4.6. HPLC-DAD-ESI-MS Analysis

Phytochemical fingerprinting of the polyphenolic fraction was performed using an Agilent 1200 HPLC system equipped with a quaternary pump, online degasser, autosampler, and diode array detector (DAD), coupled to a single-quadrupole mass spectrometer (Agilent model 6110; Agilent Technologies, Santa Clara, CA, USA) [8].

Chromatographic separation was achieved on a Kinetex XB C18 column (150 mm × 4.6 mm, 5 µm particle size; Phenomenex, Torrance, CA, USA), maintained at 25 °C, using a mobile phase consisting of (A) water containing 0.1% acetic acid and (B) acetonitrile containing 0.1% acetic acid, at a flow rate of 0.5 mL/min. The following gradient elution program was applied (expressed as % B, *v*/*v*): 0 min, 5%; 0–2 min, 5%; 2–18 min, 5 → 40%; 18–20 min, 40 → 90%; 20–24 min, 90%; 24–25 min, 90 → 5%; 25–30 min, 5% (total run time: 30 min) [15].

UV-Vis spectral data were acquired over the 200–600 nm range for all detected peaks; extracted chromatograms are reported at λ = 340. Mass spectrometric detection was performed in positive electrospray ionization (ESI^+^) full-scan mode, under the following conditions: capillary voltage, 3000 V; source temperature, 350 °C; nitrogen (drying gas) flow rate, 7 L/min; and scan range, *m*/*z* 120–1200 [8,32]. Data acquisition and processing were performed using Agilent ChemStation software (Rev. B.02.01-SR2). Negative-ion ESI was not systematically evaluated during method development; therefore, no complementary ESI^−^ dataset was available for structural annotation. The chromatographic method was intended primarily for comparative profiling of the major detectable phenolic constituents rather than for exhaustive metabolomic characterization.

### 4.7. Identification and Tentative Characterization of Phenolic Compounds

Phenolic compounds were identified or tentatively characterized according to the available analytical evidence. Compounds for which authentic reference standards were available were identified by comparing their retention times, UV-Vis absorption spectra, and mass spectral data with those of the corresponding standards analyzed under the same chromatographic conditions. Compounds showing agreement with authentic standards were considered confirmed. In the absence of authentic standards, chromatographic peaks were tentatively annotated based on their retention behavior, UV-Vis spectral characteristics, the observed protonated molecular ions ([M + H]^+^), and the aglycone ions produced by in-source fragmentation, together with comparison with previously published HPLC-DAD-MS data for *Polygonum aviculare* L. and related *Polygonum* taxa [8,15,32]. Because the single-quadrupole mass spectrometer was operated in ESI^+^ full-scan mode and no high-resolution accurate-mass MS/MS data were available, these assignments were considered tentative and were not regarded as unequivocal with respect to glycosylation position or certain structural and sugar isomers. Specific assignments such as avicularin, isoquercitrin, and quercitrin were therefore retained as plausible candidates based on their agreement with previously reported constituents of *P. aviculare*. The Phenol-Explorer database was additionally consulted for compound nomenclature and previously reported occurrence [33]. Identification confidence was assigned according to the recommendations of the Metabolomics Standards Initiative [34].

### 4.8. Calibration and Quantification

External calibration curves were constructed using chlorogenic acid and rutin as reference standards. The standards were dissolved in methanol and diluted to obtain five calibration levels for chlorogenic acid (10, 25, 30, 40, and 50 µg/mL) and six calibration levels for rutin (10, 20, 40, 60, 80, and 100 µg/mL). Calibration curves were generated by plotting the chromatographic peak area (*y*) against standard concentration (*x*, µg/mL). Linear regression yielded the equations *y* = 22.585*x* − 36.728 (R^2^ = 0.9937) for chlorogenic acid and *y* = 26.935*x* − 33.784 (R^2^ = 0.9981) for rutin. The LOD and LOQ values were 0.41 and 1.64 µg/mL, respectively, for chlorogenic acid, and 0.21 and 0.84 µg/mL, respectively, for rutin [35]. The source method reported these LOD and LOQ values but did not specify whether they were derived from calibration-curve statistics or signal-to-noise criteria. Additional validation parameters, including recovery, repeatability, sample stability, and method precision, were not available for the present dataset and therefore could not be retrospectively assessed. Samples exceeding the upper calibration limits were diluted prior to injection, and the corresponding dilution factor was included in the final calculations. All determinations were performed within the established calibration ranges. Hydroxycinnamic acids were quantified using the chlorogenic acid calibration curve and expressed as chlorogenic acid equivalents, whereas flavonols were estimated using the rutin calibration curve and expressed as rutin equivalents [8,15]. Rutin was selected as a surrogate standard because the majority of the detected flavonols were glycosylated derivatives; compounds lacking authentic standards were therefore semi-quantitatively estimated and expressed as rutin equivalents (RE). The results were reported as mg equivalents/g dry weight (dw).

### 4.9. Statistical Analysis

Three independent plants per population were treated as biological replicates (*n* = 3). Technical chromatographic replicate measurements were averaged before statistical analysis, and results are expressed as mean ± standard deviation (SD). Differences in the concentrations of individual phenolic compounds and phenolic subclasses among the five populations (PAV1–PAV5) were evaluated using one-way analysis of variance (ANOVA), followed by Tukey’s honestly significant difference (HSD) post hoc test for multiple comparisons. In the tables, different lowercase superscript letters within the same row indicate statistically significant differences among populations (*p* < 0.05). The relationship between collection-site altitude and phenolic content was assessed using Spearman rank correlation analysis, applied to the chromatographic sum of quantified phenolic compounds and to the four phenolic subclasses. One-way ANOVA, Tukey’s HSD post hoc tests, and correlation analyses were performed using JASP software, version 0.18 (JASP Team. Computer software. University of Amsterdam, Amsterdam, The Netherlands.). To visualize the relative distribution of individual phenolic compounds across populations independently of their absolute concentrations, row-wise standardized values (z-scores) were computed and represented as a heatmap generated in MATLAB, version R2023b (The MathWorks, Inc., Natick, MA, USA). Hierarchical cluster analysis was additionally performed on the standardized phenolic data using Euclidean distance and Ward’s linkage method to assess similarities among populations. Differences were considered statistically significant at *p* < 0.05.

## 5. Conclusions

This study provides the first pharmacobotanical and phytochemical characterization of PAV populations from Bihor County, northwestern Romania. Macroscopic and microscopic examination confirmed the botanical identity of the collected material and revealed a consistent anatomical organization across populations, alongside a qualitative morphological gradient related to collection altitude. HPLC-DAD-ESI-MS analysis identified fourteen phenolic compounds, dominated by flavonol glycosides of quercetin and kaempferol, with avicularin tentatively identified as a candidate phytochemical marker across all populations. The chromatographically estimated phenolic levels varied markedly among populations, with an approximately six-fold difference in the chromatographic sum between the populations showing the highest and lowest quantified values, while the qualitative fingerprint remained conserved. Exploratory statistical analysis revealed a general inverse tendency between the environmental gradient and phenolic content; however, the chromatographic sum of quantified phenolic compounds was not significantly associated with altitude, and only the hydroxycinnamic fraction (chlorogenic acid) showed a nominally significant negative correlation. These results should therefore be regarded as exploratory and cannot be attributed to altitude alone, since altitude co-varied with temperature, precipitation, and soil type. The pronounced inter-population variability documented here underscores that wild-harvested PAV cannot be assumed to be of uniform composition; consequently, the use of standardized (titrated) extracts, in which a defined marker such as avicularin or the total flavonol content is adjusted to a fixed value, is recommended to ensure reproducible quality of PAV-based preparations. Overall, these findings contribute baseline pharmacobotanical and phytochemical data for wild PAV from Romania, support its potential as a source of bioactive polyphenols, and highlight the importance of combining botanical authentication with quantitative phenolic profiling and extract standardization for the future pharmaceutical valorization of this herbal raw material.

## Figures and Tables

**Table 1:** Chromatographic and spectrometric characteristics of the identified phenolic compounds.

No.	Compound	Subclass	R_t_ (min)	UV λ_max_ (nm)	[M + H]^+^ (*m*/*z*)	Identification Basis/Confidence
**1**	Chlorogenic acid	Hydroxycinnamic acid	11.58	330	355	Authentic standard; confirmed
**2**	Myricetin-O-glucuronide	Flavonol glycoside	14.37	360, 262	495, 319	UV-Vis + MS + literature; tentative
**3**	Myricetin-O-glucoside		14.85	360, 262	481, 319	UV-Vis + MS + literature; tentative
**4**	Quercetin-3-O-rutinoside (rutin)		15.05	360, 250	611, 303	Authentic standard; confirmed
**5**	Quercetin-O-glucuronide		15.43	360, 250	479, 303	UV-Vis + MS + literature; tentative
**6**	Quercetin-O-arabinoside (tentatively assigned as avicularin)		15.56	360, 250	435, 303	UV-Vis + MS + literature; tentative
**7**	Quercetin-O-glucoside (tentatively assigned as isoquercitrin)		15.73	360, 250	465, 303	UV-Vis + MS + literature; tentative
**8**	Quercetin-O-rhamnoside (tentatively assigned as quercitrin)		16.53	360, 250	449, 303	UV-Vis + MS + literature; tentative
**9**	Kaempferol-O-glucuronide		16.62	350, 260	463, 287	UV-Vis + MS + literature; tentative
**10**	Kaempferol-O-glucoside		16.96	350, 260	449, 287	UV-Vis + MS + literature; tentative
**11**	Kaempferol-O-arabinoside		17.96	350, 260	419, 287	UV-Vis + MS + literature; tentative
**12**	Quercetin-O-galloylglucoside		18.21	360, 250	617, 303	UV-Vis + MS + literature; tentative
**13**	Kaempferol-O-malonylglucoside		19.34	350, 260	535, 287	UV-Vis + MS + literature; tentative
**14**	Kaempferol	Flavonol (aglycone)	22.08	350, 260	287	UV-Vis + MS + literature; tentative

Compounds confirmed with authentic reference standards are indicated as “confirmed”; all other compound assignments are tentative and were based on retention behavior, UV-Vis characteristics, MS data, and comparison with published literature.

**Table 2:** Chromatographically estimated contents of individual phenolic compounds in PAV populations.

No.	Compound	Abbreviation	PAV1	PAV2	PAV3	PAV4	PAV5
**1**	Chlorogenic acid	CGA	1.30 ± 0.02 ^d^	0.55 ± 0.01 ^c^	0.60 ± 0.02 ^c^	0.44 ± 0.02 ^b^	0.12 ± 0.02 ^a^
**2**	Myricetin-O-glucuronide	Myr-GlcA	0.32 ± 0.01 ^b^	0.39 ± 0.02 ^c^	0.81 ± 0.02 ^d^	0.29 ± 0.02 ^b^	0.12 ± 0.02 ^a^
**3**	Myricetin-O-glucoside	Myr-Glc	0.33 ± 0.02 ^d^	0.30 ± 0.02 ^c^	0.22 ± 0.02 ^b^	0.28 ± 0.02 ^c^	0.05 ± 0.02 ^a^
**4**	Quercetin-3-O-rutinoside (rutin)	RUT	0.41 ± 0.02 ^d^	0.17 ± 0.02 ^b^	0.22 ± 0.02 ^b^	0.23 ± 0.02 ^c^	0.04 ± 0.02 ^a^
**5**	Quercetin-O-glucuronide	Que-GlcA	0.99 ± 0.02 ^d^	0.44 ± 0.02 ^b^	0.48 ± 0.02 ^b^	0.63 ± 0.02 ^c^	0.15 ± 0.02 ^a^
**6**	Quercetin-O-arabinoside	Que-Ara	2.05 ± 0.02 ^d^	1.68 ± 0.02 ^b^	1.69 ± 0.02 ^b^	1.81 ± 0.02 ^c^	0.65 ± 0.02 ^a^
**7**	Quercetin-O-glucoside	Que-Glc	0.95 ± 0.02 ^d^	0.64 ± 0.02 ^c^	0.65 ± 0.02 ^c^	0.47 ± 0.02 ^b^	0.19 ± 0.02 ^a^
**8**	Quercetin-O-rhamnoside	Que-Rha	1.27 ± 0.03 ^e^	0.43 ± 0.02 ^b^	0.50 ± 0.02 ^c^	1.00 ± 0.02 ^d^	0.18 ± 0.02 ^a^
**9**	Kaempferol-O-glucuronide	Kae-GlcA	2.61 ± 0.02 ^d^	1.85 ± 0.01 ^b^	1.89 ± 0.01 ^c^	2.65 ± 0.01 ^e^	0.69 ± 0.01 ^a^
**10**	Kaempferol-O-glucoside	Kae-Glc	1.92 ± 0.02 ^e^	0.85 ± 0.02 ^b^	1.20 ± 0.02 ^d^	0.95 ± 0.02 ^c^	0.26 ± 0.02 ^a^
**11**	Kaempferol-O-arabinoside	Kae-Ara	0.12 ± 0.02 ^b^	0.05 ± 0.01 ^a^	0.14 ± 0.01 ^b^	0.07 ± 0.01 ^a^	0.04 ± 0.01 ^a^
**12**	Quercetin-O-galloylglucoside	Que-Gall-Glc	0.80 ± 0.02 ^d^	0.35 ± 0.02 ^b^	0.97 ± 0.02 ^e^	0.58 ± 0.02 ^c^	0.21 ± 0.02 ^a^
**13**	Kaempferol-O-malonylglucoside	Kae-Mal-Glc	5.59 ± 0.02 ^e^	1.95 ± 0.02 ^c^	1.83 ± 0.02 ^b^	4.68 ± 0.02 ^d^	0.34 ± 0.02 ^a^
**14**	Kaempferol	Kae	0.16 ± 0.02 ^a^	0.15 ± 0.02 ^a^	0.17 ± 0.02 ^b^	0.14 ± 0.02 ^a^	0.11 ± 0.02 ^a^

Note: Values are expressed as mean ± standard deviation (SD) of three independent biological replicates (*n* = 3), in mg/g dry weight (dw). The SD reflects variability among the biological replicates and not analytical method precision. Chlorogenic acid and rutin were quantified using authentic reference standards and are reported as mg chlorogenic acid/g dw and mg rutin/g dw, respectively; all other flavonol derivatives were semi-quantitatively estimated using the rutin calibration curve and are expressed as mg rutin equivalents (RE)/g dw. Different superscript letters within the same row indicate significant differences among populations (Tukey’s HSD, *p* < 0.05). Compounds **6**–**8** were tentatively assigned as avicularin, isoquercitrin, and quercitrin, respectively. Detailed data are presented in Supplementary Table S1.

**Table 3:** Distribution of the chromatographically quantified phenolic subclasses in the five PAV populations (mg/g dry weight; percentage of the chromatographic sum of quantified phenolic compounds).

Phenolic Subclass	PAV1	PAV2	PAV3	PAV4	PAV5
Hydroxycinnamic acid (mg CAE/g dw)	1.30 ± 0.02	0.55 ± 0.02	0.60 ± 0.02	0.44 ± 0.02	0.12 ± 0.02
Hydroxycinnamic acid (% of total)	6.9	5.6	5.2	3.1	3.8
Quercetin glycosides (mg RE/g dw)	6.47 ± 0.06	3.71 ± 0.05	4.50 ± 0.05	4.72 ± 0.05	1.42 ± 0.05
Quercetin glycosides (% of total)	34.4	37.9	39.6	33.2	45.2
Kaempferol derivatives (mg RE/g dw)	10.40 ± 0.04	4.84 ± 0.04	5.23 ± 0.04	8.48 ± 0.04	1.43 ± 0.04
Kaempferol derivatives (% of total)	55.3	49.4	46.0	59.6	45.7
Myricetin glycosides (mg RE/g dw)	0.64 ± 0.02	0.69 ± 0.02	1.03 ± 0.02	0.57 ± 0.02	0.17 ± 0.02
Myricetin glycosides (% of total)	3.4	7.0	9.1	4.0	5.3
Chromatographic sum of quantified compounds (mg/g dw)	18.81 ± 0.08	9.79 ± 0.07	11.36 ± 0.07	14.22 ± 0.07	3.14 ± 0.07
Dominant compound	Kaempferol-O-malonylglucoside	Kaempferol-O-malonylglucoside	Kaempferol-O-glucuronide	Kaempferol-O-malonylglucoside	Kaempferol-O-glucuronide

Values are expressed as mean ± standard deviation (*n* = 3), in mg/g dry weight. CAE = chlorogenic acid equivalents; RE = rutin equivalents. The flavonol glycosides listed in Table 2 are grouped according to their aglycone moiety; free kaempferol (aglycone) is included within the kaempferol group.

**Table 4:** Correlations between altitude and chromatographic sum of quantified phenolic compounds in the *Polygonum aviculare* L. populations.

Parameter	Spearman ρ	*p*
Chromatographic sum of quantified phenolic compounds	−0.600	0.285
Hydroxycinnamic acids	−0.900	0.037 *
Quercetin glycosides	−0.600	0.285
Kaempferol derivatives	−0.600	0.285
Myricetin glycosides	−0.600	0.285

Spearman rank correlation coefficients between collection-site altitude (105–605 m a.s.l.) and the chromatographic sum of quantified phenolic compounds and phenolic subclasses, across the five populations (*n* = 5). * *p* < 0.05. The chromatographic sum of quantified phenolic compounds was not significantly correlated with altitude. Given the small number of populations and the covariation of altitude with temperature, precipitation, and soil type, these correlations are exploratory and do not establish altitude as an independent driver.

**Table 5:** Sampling sites, geographic and pedoclimatic characteristics of the collected *Polygonum aviculare* L. populations. Pedoclimatic characteristics were compiled from regional literature and publicly available climatic and soil datasets [26,27,28].

Code	Location	Altitude	Coordinates	Geographic Zone	Mean Annual Temp.	Annual Precipitation	Predominant Regional Soil Class
PAV1	Santăul Mare	105 m	47°10′03″ N 21°50′20″ E	Câmpia Crișurilor (plain)	~10.5 °C	~600 mm	Alluvial soils/chernozems
PAV2	Șoimi	201 m	46°40′29″ N 22°06′43″ E	Codru-Moma piedmont (hilly)	~9–10 °C	~650 mm	Cambisols (luvisols)
PAV3	Dumbrăvița de Codru	380 m	46°39′17″ N 22°10′35″ E	Codru-Moma (upper hilly)	~8–9 °C	~700–750 mm	Cambisols
PAV4	Izbuc	515 m	46°26′17″ N 22°27′32″ E	Apuseni Mts., karst (Pădurea Craiului)	~7–8 °C	~900–1000 mm	Rendzic Leptosols (limestone)
PAV5	Băița	605 m	46°28′34″ N 22°35′10″ E	Apuseni Mts. (Bihor Mts.), karst	~7–7.5 °C	~1000 mm	Rendzic Leptosols/mtn. cambisols

Note: Pedoclimatic characteristics, including predominant regional soil classes, were compiled from regional literature and publicly available climatic and soil datasets. No direct physicochemical soil analyses were performed at the sampling sites. Soil class was included as contextual pedoclimatic information and was not treated as an independent explanatory variable in the statistical analysis.

## Data Availability

The original contributions presented in the study are included in the article/Supplementary Materials. Further inquiries can be directed to the corresponding author.

## Supplementary Materials

The following supporting information can be downloaded at: https://www.mdpi.com/article/10.3390/molecules31183237/s1, Table S1: Individual quantitative values of the three biological replicates for the identified phenolic compounds in the five *Polygonum aviculare* populations.

## Abbreviations

The following abbreviations are used in this manuscript:

Abbreviation	Definition
ANOVA	Analysis of variance
CAE	Chlorogenic acid equivalents
CGA	Chlorogenic acid
DAD	Diode array detector
dw	Dry weight
ESI	Electrospray ionization
ESI^+^	Positive electrospray ionization
HPLC	High-performance liquid chromatography
HPLC-DAD	High-performance liquid chromatography coupled with diode array detection
HPLC-DAD-ESI-MS	High-performance liquid chromatography–diode array detection–electrospray ionization mass spectrometry
HSD	Honestly significant difference
Kae	Kaempferol
Kae-Ara	Kaempferol-O-arabinoside
Kae-Glc	Kaempferol-O-glucoside
Kae-GlcA	Kaempferol-O-glucuronide
Kae-Mal-Glc	Kaempferol-O-malonylglucoside
LOD	Limit of detection
LOQ	Limit of quantification
MS	Mass spectrometry
MSI	Metabolomics Standards Initiative
Myr-Glc	Myricetin-O-glucoside
Myr-GlcA	Myricetin-O-glucuronide
PAV	*Polygonum aviculare* L.
PAV1	*Polygonum aviculare* L. population from Santăul Mare (105 m a.s.l.)
PAV2	*Polygonum aviculare* L. population from Șoimi (201 m a.s.l.)
PAV3	*Polygonum aviculare* L. population from Dumbrăvița de Codru (380 m a.s.l.)
PAV4	*Polygonum aviculare* L. population from Izbuc (515 m a.s.l.)
PAV5	*Polygonum aviculare* L. population from Băița (605 m a.s.l.)
Que-Ara	Quercetin-O-arabinoside
Que-Gall-Glc	Quercetin-O-galloylglucoside
Que-Glc	Quercetin-O-glucoside
Que-GlcA	Quercetin-O-glucuronide
Que-Rha	Quercetin-O-rhamnoside
RE	Rutin equivalents
RUT	Rutin
SD	Standard deviation
UV-Vis	Ultraviolet–visible spectroscopy
